# Livin in synergy with Ras induces and sustains corticosteroid resistance in the airway mucosa

**DOI:** 10.7150/ijbs.58427

**Published:** 2021-05-13

**Authors:** Jin-Mei Xue, Yun-Fang An, Li-Min Suo, Li-Hua Mo, Gui Yang, Xiang-Qian Luo, Da-Bo Liu, Chang-Qing Zhao, Ping-Chang Yang

**Affiliations:** 1 Department of Otolaryngology, Head & Neck Surgery, Second Hospital, Shanxi Medical University, Taiyuan, China.; 2 Guangdong Provincial Key Laboratory of Regional Immunity and Diseases, Shenzhen, China.; 3 Research Center of Allergy & Immunology, Shenzhen University School of Medicine, Shenzhen, China.; 4 Department of Otolaryngology, Longgang Central Hospital, Shenzhen, China.; 5 Department of Pediatric Otolaryngology, Shenzhen Hospital, Southern Medical University, Shenzhen, China.

**Keywords:** nasal mucosa, epithelial cell, inflammation, corticosteroid resistance, glucocorticoid receptor, livin

## Abstract

**Rationale**: Corticosteroid resistance (CR) seriously affects the therapeutic effects of steroids on many chronic inflammatory disorders, including airway allergy. The mechanism of CR development is unclear. Recent research indicates that livin, an apoptosis inhibitor, is associated with the regulation in cell activities. This study investigates the role of livin in the inducing and sustaining CR in the airway mucosa.

**Methods**: Nasal epithelial cells (NECs) were isolated from surgically removed nasal mucosal tissues of patients with allergic rhinitis (AR) and nasal polyps with or without CR. Differentially expressed genes in NECs were analyzed by the RNA sequencing. A CR mouse model was developed to test the role of livin in CR development.

**Results**: The results showed that NECs of AR patients with CR expressed high levels of livin, that was positively correlated with the thymic stromal lymphopoietin (TSLP) expression and the high Ras activation status in NECs. Livin and Ras activation mutually potentiating each other in the inducing and sustaining the TSLP expression in NECs. TSLP induced eosinophils and neutrophils to express glucocorticoid receptor-β (GRβ). Eosinophils and neutrophils with high CRβ expression were resistant to corticosteroids. Depletion of livin or inhibition of TSLP markedly attenuated CR and airway allergy.

**Conclusions**: Livin facilitates CR development in the airways by promoting TSLP expression in epithelial cells and the GRβ expression in eosinophils and neutrophils. Depletion of livin or inhibiting TSLP attenuates CR development and inhibits airway allergy, this has the translational potential to be used in the treatment of airway allergy.

## Introduction

There are many diseases, e.g., chronic pulmonary diseases, chronic kidney diseases, inflammatory bowel diseases, rheumatoid arthritis, rely on the steroid therapy that has strong effects on inhibiting inflammation non-specifically 1. The steroid therapy usually takes a long time, or lifetime in some patients, on the purpose of symptom control. Among these patients, up to 30% do not respond to moderate doses of corticosteroids, a condition designated corticosteroid resistance (CR) 2. As CR results in inadequate disease control; consequently, the inflammatory condition continues, that induces tissue damage, steroid associated side-effects, e.g., weight gain, osteoporosis, impaired glucose tolerance and hypertension 3. Yet, the mechanism and causative factors of CR development are not fully understood.

The intracellular glucocorticoid receptor-α (GRα) and GRβ mediate the effects of steroids on regulating cell activities 3. Steroids diffuse into the cytoplasm to bind and activate GRα; the latter then translocates into the nucleus to exert its biological activities 3. GRβ is a transcript of the same gene of GRα, but different splicing. GRβ does not mediate the steroid effects on regulating nuclear activities, but competitively interferes with the binding between GRα and steroids. Thus, GRβ exerts inhibitory effects of steroids 3. It is the consensus that GRβ over expression is the canonical factor in the CR development 4. Although it is known that almost all body cells express GRβ 4, the mechanism of GRβ over expression still remains elusive.

CR is common in chronic airway diseases, including severe asthma, chronic obstructive pulmonary disease, nasal allergy (AR) and chronic rhinosinusitis 3, 5, 6. To find hints to elucidate the pathogenesis of CR, we collected nasal epithelial cells (NECs) of patients with nasal allergy, nasal polyposis with or without CR. By RNA sequencing (RNAseq), we found that the livin gene was more active among the differentially expressed genes in CR NECs. Livin is one of the apoptosis inhibitors and is found in many cancers 7. Livin promotes cancer growth 7, activates dendritic cells and CD8^+^ cytotoxic lymphocytes 8. Based on the above information, we hypothesize that livin may be involved in the CR development. To test the hypothesis, we collected surgically removed nasal mucosal tissues from AR patients with nasal polyps and chronic rhinosinusitis with or without CR. The association between the expression of livin and thymic stromal lymphopoietin (TSLP) in nasal epithelial cells and the CR development was investigated.

## Materials and methods

### Human subjects

Patients with chronic rhinosinusitis, nasal allergy and polyps were recruited into this study. The patients were divided into two groups, corticosteroid resistant (CR) group and corticosteroid sensitive (CS) group based on reported criteria 9. Nasal polyps were diagnosed based on endoscopic exam findings of nasal polyps from the middle nasal meatus. CT scan was performed for each patient to determine the nasal polyp size and rhinosinusitis. Nasal allergy was diagnosed based on the disease history, antigen skin prick test (SPT) (Table S1) and serum specific IgE assessment. Chronic rhinosinusitis was diagnosed based on the disease history, nasal exam and CT scan findings. The diagnosis and management of these patients were carried out by doctors in our department following the established procedures that can be found elsewhere 9. The demographic data are presented in Table 1. The experimental procedures were approved by the Human Ethical Committee at Shanxi Medical University (#SMUETHHU2018012). An informed written consent was obtained from each human subject.

### Nasal polyp scoring

Following published data 9, nasal polyps were scored for each patient. This is a 5-point scoring system including: 0, No polyps; 1, small polyps in the middle meatus not reaching below the inferior border of the middle concha; 2, polyps reaching below the lower border of the middle turbinate; 3, large polyps reaching the lower border of the attachment of inferior turbinate or polyps medial to the middle concha and 4, large polyps causing almost complete congestion/obstruction of the inferior meatus.

### Determining CR and CS status in patients with nasal polyp, nasal allergy and chronic rhinosinusitis

All patients were not used corticosteroid agents, immune suppressive agents and anti-allergy agents at least one month prior to the recruitment. To determine whether the patients were at the CR status, following established procedures 6, patients were prescribed with oral corticosteroids (30 mg of prednisone once daily for 14 days). Polyp scores were assessed before and after the treatment with oral corticosteroids; subjects with polyp size reduced ≤ 1 score were categorized to the CR (CR) group, otherwise the CS group (Table S2).

### CR airway allergy mouse model development

Following published procedures 5 with minor modification, mice were sensitized by subcutaneous injection on the back skin with ovalbumin (OVA, 100 µg/mouse) mixed in 0.1 ml complete Freund adjuvant on day 0 and day 7. Between day 14 and day 21, mice were treated with nasal instillation (50 µl/nostril) containing OVA (5 mg/ml in incomplete Freund adjuvant) daily. Mice were sacrificed on day 22.

### Traditional (TR) airway allergy mouse model development

Following our established protocols 10, mice were sensitized by subcutaneous injection on the back skin with ovalbumin (OVA, 100 µg/mouse) mixed in 0.1 ml alum on day 0 and day 7. Between day 14 and day 21, mice were treated with nasal instillation (50 µl/nostril) containing OVA (5 mg/ml in saline) daily. Mice were sacrificed on day 22.

### Statistics

Each group consisted of 6 mice. Each experiment was repeated at least 3 times with each sample analyzed in triplicate (average of the three readouts was used as one datum). The data are presented as mean ± SEM. The difference between two groups was determined by the Student *t*-test (if the data are normal distributed) or the Mann Whitney test (if the data are non-normal distribution). ANOVA followed by the Bonferroni test or Dunnett's test was performed for multiple comparisons. The correlation between two data sets was determined by the Pearson correlation test (in the data of normal distribution) or the Spearman correlation test (in the data of non-normal distribution).

Some experimental procedures are presented in the supplementary materials.

## Results

### Livin expression is positively correlated with TSLP in CR NECs

**Figure 1:**
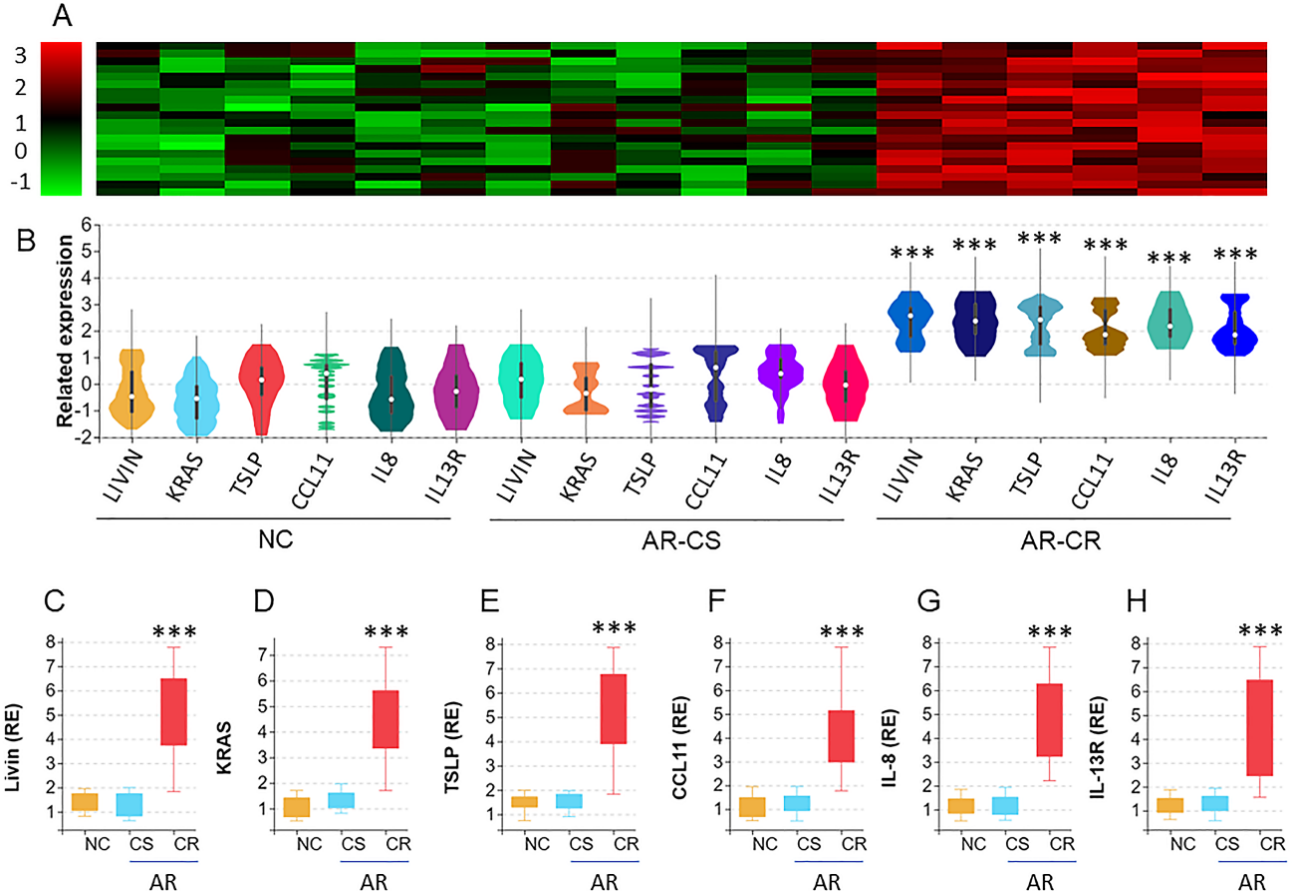
** RNA sequencing analysis of the nasal epithelial cells of AR patients with corticosteroid resistance (CR)**. A-B, nasal epithelial cell (NEC) samples were collected from patients with chronic sinusitis and polyps with CR (CR group; n=20) or without CR (CS group; n=20) and normal controls (NC; n=20). NECs were analyzed by RNAseq. The heatmap and violin plots shows 6 differentially expressed genes (DEG). C-H, bars show the DEG mRNA levels of panel B (by RT-qPCR; RE: related expression). ***, p<0.001 (the Mann Whitney test). The error bars show data range. Samples from individual patients were processed separately. Each sample was tested in triplicate.

We collected surgically removed nasal mucosa from AR-polyp patients with CR or without CR (CS). Nasal epithelial cells (NEC) were isolated from the samples by enzymatic digestion and flow cytometry cell sorting (FCS) and analyzed by the RNAseq. The results revealed 6 differentially expressed genes (DEG), including Livin, KRAS (hereafter, Ras, in short), TSLP, CCL11, IL-8 and IL-13R, in nasal epithelial cells. The DEG levels were significantly higher in AR-CR samples than that in NC samples and AR-CS samples. No statistical difference was detected between the AR-CS group and the NC group (Fig. 1A-B). The RNAseq results were verified by conventional RT-qPCR (Fig. 1C-H). The results also showed positive correlation between livin and Ras or TSLP in AR-CR epithelial cells, but not in AR-CS or NC epithelial cells (Fig. S1 in supplemental materials. The results suggest that the activities of livin, Ras and TSLP in NECs may be associated with the CR status in the nasal mucosa.

### Ras activation is involved in the expression of livin and TSLP in NECs

**Figure 2:**
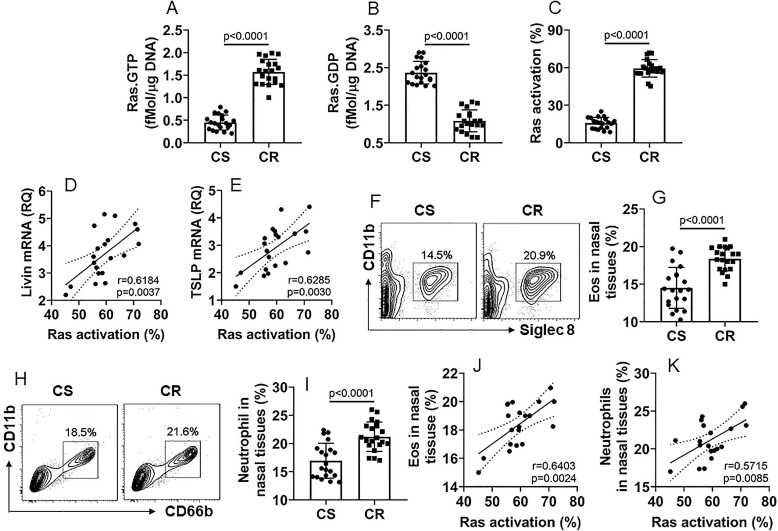
** Ras activation is associated with the expression of livin and TSLP in CR NECs**. A-C, NECs were isolated from the surgically removed CS (n=20) and CR (n=20) nasal tissues as described in Fig. 1 and analyzed by Ras-specific ELISA. The bars indicate the levels of Ras.GTP (A), Ras.GDP (B) and Ras activation (C) in NECs. D-E, scatter plots show positive correlation between Ras activation and livin mRNA levels (D) or TSLP mRNA levels (E) in NECs (the data of livin and TSLP mRNA are presented in Fig. 1), F-I, mononuclear cells were isolated from the nasal tissues and analyzed by FCS. Gated plots show Eo counts (F) and neutrophil counts (H). Bars show summarized Eo counts (G) and neutrophil counts (I). J-K, scatter plots show positive correlation between Ras activation and Eo counts (J) or neutrophil counts (K) in nasal tissue-isolated mononuclear cells. Statistics: the Mann Whitney test (A-C, G, I) and the Spearman correlation test (D, E, J, K). Samples from individual patients were analyzed separately. Each dot in bars present data obtained from one sample.

Prompted by the results of Ras levels were higher in CR NECs, we inferred that Ras activation might also be involved in promoting the livin and TSLP expression in CR NECs. To this end, we analyzed Ras activation in NECs by the Ras-specific ELISA. The results showed that, compared to the CS group, Ras activation was significantly higher in NECs of the CR group (Fig. 2A-C). Positive correlation was detected between the Ras activation and the livin expression or the TSLP expression, in CR NECs (Fig. 2D-E). Furthermore, we counted eosinophils (Eo) and neutrophils in the nasal mucosa; the Eo counts and neutrophil counts were higher in the CR group than those in the CS group (Fig. 2F-I). The Eo counts and neutrophil counts in the CR group were also positively correlated with Ras activation in NECs (Fig. 2J-K). The results suggest that the Ras activation may be associated with the expression of livin and TSLP, as well as the infiltration of Eo and neutrophils in the CR nasal mucosa.

### Livin mediates the effects of Ras activation on inducing TSLP expression in NECs

**Figure 3:**
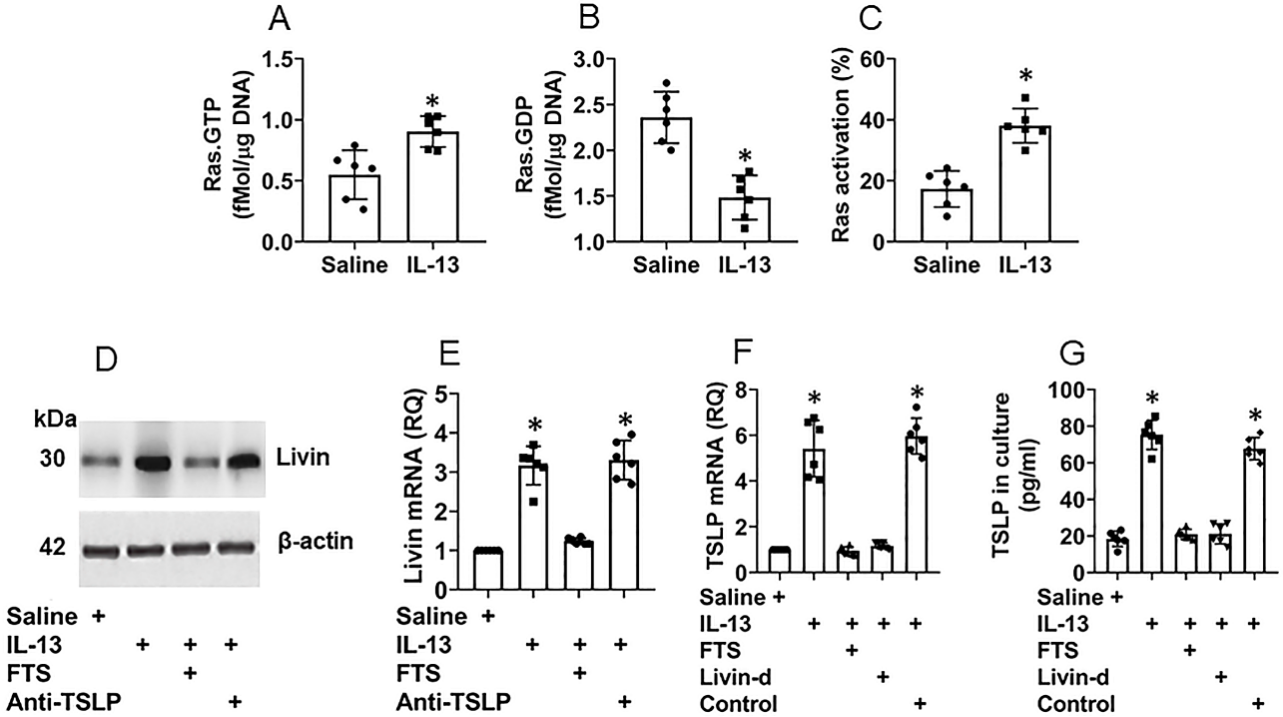
** Ras activation and livin are involved in TSLP expression in NECs**. Naive NECs were isolated from surgically removed nasal tissues of patients with nasal cancer (n=6). The adjacent normal nasal tissues were isolated (proved by a pathologist) to be used in the experiments. NECs were cultured in the presence of agents denoted below the bar graphs. IL-13 (10 ng/ml), FTS (a Ras inhibitor; 75 µM). Anti-TSLP: Anti-TSLP mAb (20 ng/ml; isotype IgG also did not show any effects on livin expression; not shown). Cells were collected and analyzed 48 h later. A-C, bars show Ras.GTP (A), Ras.GDP (B) and Ras activation (C) levels in NECs. D, immunoblots show livin protein levels in NECs. E-F, bars show mRNA levels of livin (E) and TSLP (F) in NECs. G, bars show TSLP in culture supernatant. *, p<0.01, compared with the saline group. Statistical methods: The Student *t*-test (A-C) and ANOVA followed by the Dunnett's test (E-G). Each dot in bars present data obtained from individual sample. The data represent 6 independent experiments. Samples of panel D were not pooled.

Although it has been recognized that TSLP plays an important role in the CR development 11, how the TSLP expression is sustained at high levels in the CR subjects 12 remains to be investigated. Thus, we sought to elucidate the mechanism by which the TSLP expression is sustaining at high levels in NECs of CR subjects. Following the established procedures of inducing TSLP expression 13, we exposed NECs [the cells express IL-13 receptor (Fig. S2A-C)] to IL-13 in the culture for 48 h. The results showed that, in line with previous reports 13, exposure to IL-13 markedly increased the TSLP expression in NECs that was released into the culture supernatant (Fig. S2D-E). The exposure to IL-13 also induced Ras activation (Fig. 3A-C), livin expression (Fig. 3D-E) and TSLP expression (Fig. 3F-G) in NECs. Inhibition of Ras, but not TSLP, abolished the effects of IL-13 on inducing livin expression (Fig. 3D-E), indicating that IL-13 induces livin expression via activating Ras; the TSLP expression is the downstream of livin. Inhibition of either Ras or livin (Fig. S3) abolished the IL-13-induced TSLP expression (Fig. 3F-G). The results indicate that livin mediates the effects of Ras activation on inducing the TSLP expression in NECs.

### Interaction of livin and Ras sustains TSLP production in NECs

**Figure 4:**
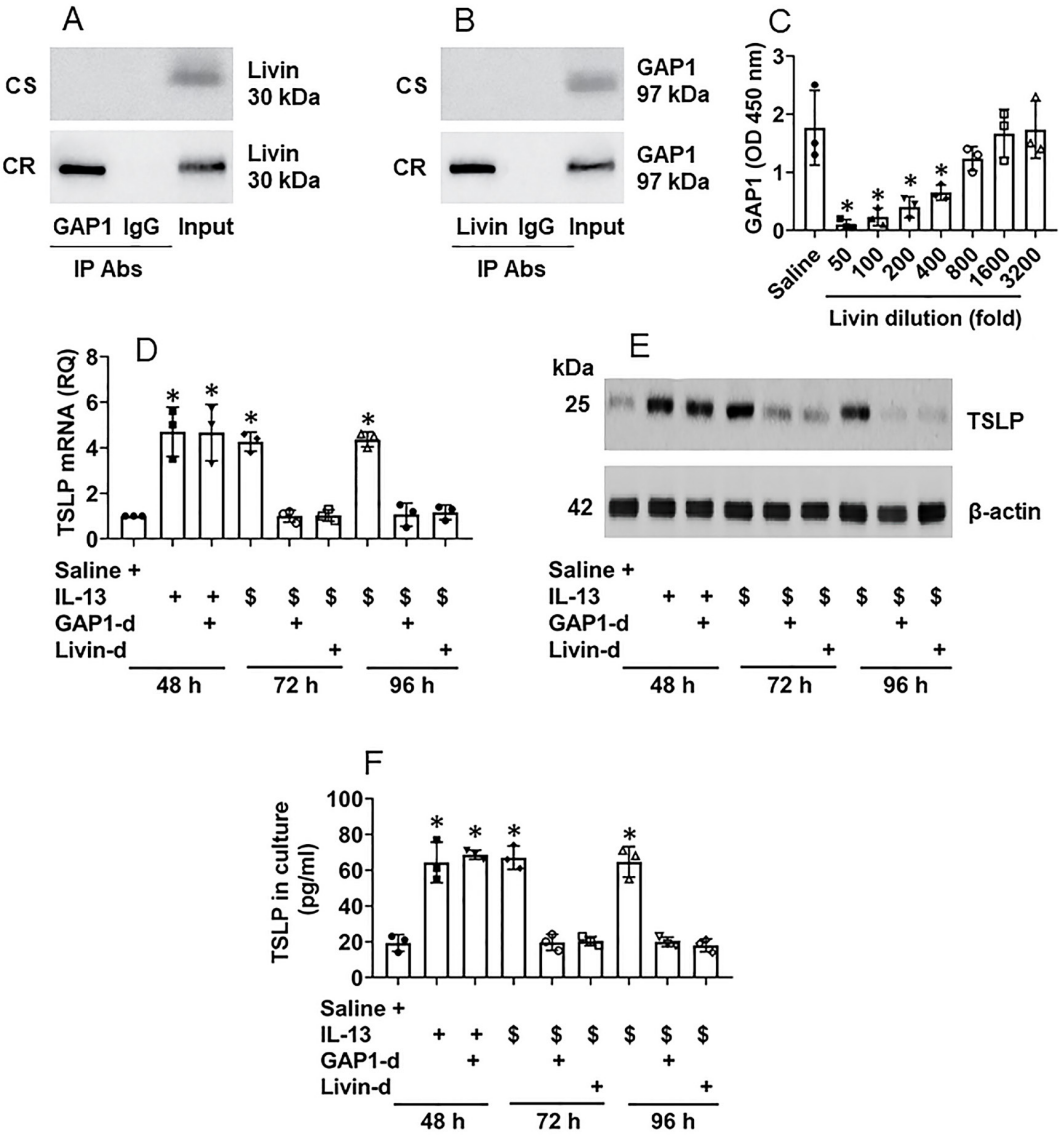
** Interaction of livin and GAP1 sustains TSLP expression in NECs**. A-B, proteins were extracted from 5 CR and 5 CS NEC samples and analyzed by co-IP. Immunoblots show a complex of GAP1 and livin in NEC extracts. C, bars show binding between Ras and GAP1 after pre-exposing GAP1 to livin at indicated concentrations (denoted on the x axis). D-F, NECs were cultured in the procedures denoted below the x axis of panel D. IL-13: 10 ng/ml in the culture. $, NECs were exposed to IL-13 in the culture for 48 h, washed with medium and continued the culture in fresh medium (without IL-13). GAP1-d: GAP-1 deficient NECs (by RNAi). Livin-d: Livin-deficient NECs (by RNAi). D, bars show TSLP mRNA levels in NECs. E, immunoblots show TSLP protein levels in NECs. F, bars show TSLP levels in culture supernatant. *, p<0.05, compared with group 0 (B) or the saline group (D, F) (ANOVA followed by the Dunnett's test). Each dot in bars present data obtained from one sample. The data represent 3 independent experiments. Samples of immunoblots were not pooled.

It is recognized that the proline-rich molecules have the capacity to bind other proteins 14. Livin is a proline-rich protein as it has four “PxxP” structures in its amino acid sequences at the sites of 79-82, 188-191, 191-194 and 199-202, respectively (Fig. S4). Therefore, by immunoprecipitation (IP) assay, we precipitated protein extracts of CR NECs with an anti-livin antibody as a bait. The precipitated complexes were analyzed by mass spectrometry (MS). The MS results showed that the complexes were composed of livin and Rac GTPase binding protein-1 (GAP1, in short) (Fig. S5). The results were verified by co-IP with either anti-livin antibody (Ab) or anti-GAP1 Ab as precipitating Abs. A complex of GAP1 and livin was detected in CR NEC extracts (Fig. 4A-B), indicating that livin can bind GAP1 to form a complex in CR NECs; such a complex was not found in CS NECs (data not shown). The results were corroborated by a competitive ELISA with recombinant proteins of livin, GAP1 and Ras. The exposure to livin competitively attenuated the binding capacity of GAP1 to Ras (Fig. 4C). As Ras activation is required in TSLP expression in CR NECs as shown by Fig. 3, GAP1 plays a critical role in the Ras deactivation 15, the physical contact between livin and GAP1 may interfere with Ras deactivation in the course of TSLP production in NECs, that may sustain the Ras activation 15 as well as sustain the TSLP expression in NECs. To test this, NECs were stimulated with IL-13 in the culture. High levels of TSLP expression could be detected at 48 h in either WT NECs, or GAP1-deficient NECs (Fig. S6) or livin (Fig. S3) expression. The NECs were then washed with culture medium and the culture was continued with fresh medium without the presence of IL-13 (the stimulant). NECs were harvested at 72 h, and 96 h, respectively. The TSLP expression was still detected in IL-13-primed WT NECs at both mRNA and protein levels (Fig. 4D-E) as well as in the culture supernatant (Fig. 4F), which was abolished by depletion of either GAP1 or livin in NECs (Fig. 4D-F). In separate experiments, NECs were exposed to IL-13 in the culture for 96 h; the TSLP expression in NECs was detected at similar levels to those NECs exposed to IL-13 for 48 h (data not shown). The results demonstrate that livin can prolong or sustain the expression of TSLP in NECs.

### NEC-derived TSLP induces GRβ expression in eosinophils and neutrophils of the airway mucosa

**Figure 5:**
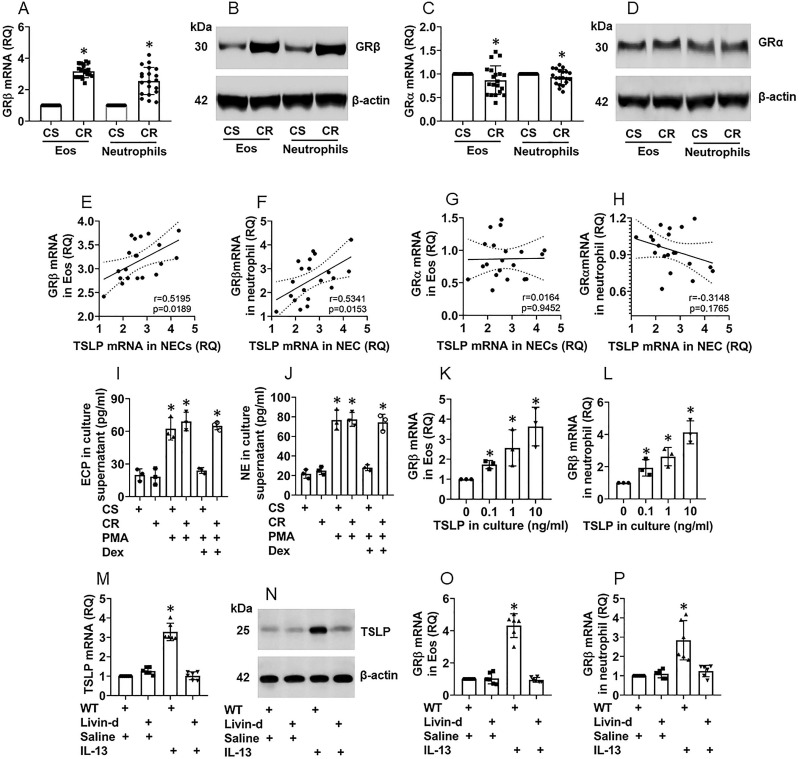
** NEC-derived TSLP induces CR in Eos and neutrophils in the nasal mucosa**. A-D, Eos and neutrophils were isolated from surgically removed nasal tissues of CR patients (n=20) and CS patients (n=20). A and C, bars show mRNA levels of GRβ (A) and GRα (C) in Eos and neutrophils. B and D, immunoblots show protein levels of GRβ (B) and GRα (D) in Eos and neutrophils. E-H, scatter plots show correlation between NEC TSLP expression and GRβ/GRα mRNA expression of Eo/neutrophils in the nasal mucosa of CR subjects. I-J, Eos and neutrophils were isolated from CR and CS nasal mucosa; the cells were activated by PMA (50 nM) in the culture. Bars show EPX (I) and NE (J) levels in culture supernatant. Dex: In the presence of dexamethasone (1 µM). K-L, Eos and neutrophils were isolated from healthy subject peripheral blood samples and stimulated by TSLP in the culture at the indicated concentrations for 48 h. Bars show GRβ mRNA levels in Eos and neutrophils. M-P, naive mice (6 per group) were treated with IL-13 solution (50 µg/ml) via nasal instillation (50 µl/nostril) daily for 7 consecutive days. Mice were sacrificed on day 8. Airway tissues (including the nasal mucosa and the lungs) were excised. Eos and neutrophils were isolated from the tissues. NECs, Eos and neutrophils were isolated from the airway tissues by FCS. Bars show TSLP mRNA levels in NECs (M), GRβ mRNA in Eos (O) and neutrophils (P); immunoblots show TSLP protein levels in NECs (N). *, p<0.01, compared to the CS group [A, C (the Student *t*-test), I, J (ANOVA followed by the Dunnett's test)], or compared to the “0” group (K, L; ANOVA followed by the Dunnett's test), or compared to the WT/saline group (M, O, P; ANOVA followed by the Dunnett's test). Statistics in E-H: Pearson correlation coefficient test. Each dot in bars present data obtained from one sample. The data represent 3 independent experiments. Samples for immunoblot experiments were not pooled.

Eosinophils (Eo) and neutrophils are inflammatory effector cells that play an important role in CR 5, 16. The overexpression of glucocorticoid receptor-β (GRβ) 17 and TSLP 11 were reported in CR subjects. Thus, we isolated Eos and neutrophils from surgically removed nasal tissues of patients with nasal polyps with or without CR. We found that, compared to CS Eos and neutrophils, the CR Eos and neutrophils expressed higher GRβ levels (Fig. 5A-B). The GRα expression was slightly lower (not reached the significant criterion) in CR Eos and neutrophils as compared to the CS Eos (Fig. 5C-D). Further analysis showed a positive correlation between the TSLP expression in NECs and the GRβ expression in Eos and neutrophils (Fig. 5E-F), but not with GRα in either Eos or neutrophils (Fig. 5G-H). Exposure of the Eos and neutrophils (isolated from the nasal tissues) to phorbol 12-myristate 13-acetate (PMA, a non-specific cell activator) in the culture activated the Eos and neutrophils manifesting a marked release of EPX (Eo peroxidase; an Eo mediator) and neutrophil elastase (NE, a neutrophil mediator) into the culture supernatant, that could be blocked by the presence of dexamethasone in CS Eos and neutrophils but not in CR Eos and neutrophils (Fig. 5I-J). The results indicate that Eos and neutrophils isolated from CR patients are resistant to corticosteroids. The results were verified by a cell culture experiment. Exposure of Eos and neutrophils (isolated from healthy subject peripheral blood samples) to TSLP in the culture markedly induced the GRβ expression in Eos and neutrophils (Fig. 5K-L, Fig. S7). Furthermore, treating mice with nasal instillation of an IL-13 solution increased the TSLP expression in NECs (Fig. 5M-N), and the GRβ expression in Eos and neutrophils in the airway tissues (Fig. 5O-P, Fig. S8). The results indicate that NEC-derived TSLP can induce GRβ expression in Eos and neutrophils and confer Eos and neutrophils the CR properties.

### Depletion of livin or blocking TSLP inhibits experimental CR in the airway mucosa

**Figure 6:**
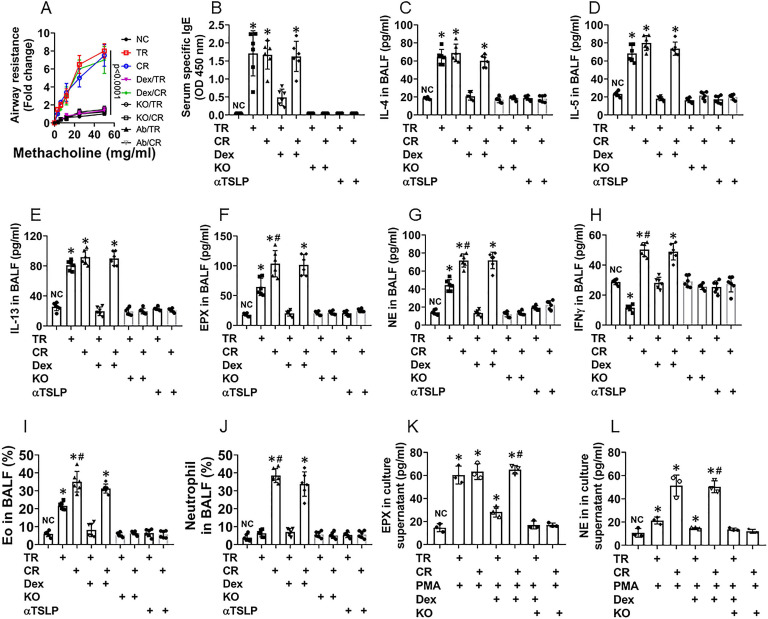
** Depletion of livin expression inhibits CR in a murine model of airway allergy**. A corticosteroid resistance (CR) airway allergy model and a traditional (TR) airway allergy murine model were developed. A, curves show mouse airway resistance in response to methacholine challenge. B, bars show serum OVA-specific IgE levels. C-H, bars show airway allergy-related cytokine levels in BALF. I-J, bars show Eo counts (J) and neutrophil counts (L) in BALF (FCS plots are presented in supplementary materials. K-L, Eos and neutrophils were isolated from BALF and treated with the procedures denoted on the x axis. Bars show EPX (M; eosinophil peroxidase; used as an Eo activation indicator) and NE (N; neutrophil elastase; used as an indicator of neutrophil activation). PMA: Phorbol 12-myristate 13-acetate (50 nM, used as an activator of Eos and neutrophils). Dex (A-H): TR mice and CR mice were treated with dexamethasone in each time of OVA exposure. Dex (I-N): Dexamethasone (1 µM) in the culture. KO mice: Mice carrying livin-deficient epithelial cells (NC mice are the littermates of KO mice). *, p<0.01, compared with the NC group (B-H, M, N) or group a (J, L; the group labels of J and L are the same as those in FCS plots on the left side) (ANOVA followed by the Bonferroni test). Each dot in bars present data obtained from one sample. Each group consists of 6 mice. Samples from individual mice were processed separately. The data represent 6 independent experiments.

Referring to established procedures 5, we developed a murine CR airway allergy murine model (CR model) as well as a traditional airway allergy murine model (TR model) 10. In line with published reference 5, CD45^+^ cells isolated from the CR mouse airway tissues showed high expression of Bcl2 (Fig. S9), indicating the CR model is successful. As shown by Fig. 6, both CR model mice (CR mice, in short) and TR mice showed airway hyper responsiveness (Fig. 6A), increase in the levels of specific IgE (Fig. 6B), the levels of IL-4, IL-5, IL-13 and EPX were increased in BALF (Fig. 6C-F), abundant Eos were found in BALF (Fig. S10). Treating TR mice and CR mice with dexamethasone at each time of OVA exposure suppressed the allergic response in the airways of TR mice, but not in CR mice (Fig. 6). Additionally, CR mice showed higher levels of neutrophil elastase (NE), IFN-γ (Fig. 6G-H) and neutrophil counts (Fig. S11) in BALF. In line with published data, in which blocking TSLP with specific antibodies inhibited experimental allergic airway Th2 inflammation 18, We found that administration of anti-TSLP antibodies blocked the allergic responses in mice treated with either traditional immunization approach or the CR-inducing approach (Fig. 6). Eos and neutrophils isolated from CR BALF showed higher GRβ levels and lower responsiveness to dexamethasone in response to the PMA challenge than those isolated from TR BALF. The results indicate that CR group mice were developed airway allergy, airway hyper responsiveness and CR, while TR mice only show airway allergy and hyper responsiveness, not CR (Fig. 6). In mice with livin-deficient epithelial cells, however, mice did not develop CR, nor airway allergy or airway hyper responsiveness (Fig. 6), that emphasizes the role of livin in the development of airway allergy and CR.

## Discussion

In this study, we found that NECs of CR subjects expressed high levels of livin, the latter cooperated with Ras activation to induce TSLP expression in NECs. CR nasal mucosa-derived Eos and neutrophils showed high levels of GRβ expression and did not respond to steroid stimulation. Exposure to TSLP induced naive Eos and neutrophils to express GRβ and develop the CR features. The findings were expanded in a murine airway allergy and the CR model study, in which the inhibition of livin or TSLP significantly attenuated airway allergic response and the CR development.

Eos and neutrophils are the canonical effector cells in airway allergy, chronic rhinosinusitis and pulmonary chronic inflammation in both CR subjects and CS subjects 5, 16. The present study showed that, not only the counts of Eo and neutrophil in the nasal tissues were significantly higher in the CR group patients than that in the CS group patients, we also found that the GRβ expression was higher in Eos and neutrophils of the CR group than that in the CS group. Published data show that CR subject peripheral leukocytes express higher GRβ levels than those of healthy subjects 19. Although GRβ is one of the receptors of corticosteroids, it does not mediate the effects of corticosteroids on attenuating inflammation, but competitively occupies the binding sites of steroid molecules, that prevents the effective receptor, GRα, to be bound by steroids. Indeed, our data show that CR Eos and neutrophils are significantly less sensitive to steroids as compared to those isolated from the CS nasal tissues. The findings were reproduced in an animal model study. Eos and neutrophils isolated from the CR group mice showed higher GRβ expression than that in the mice sensitized by traditional procedures.

To elucidate the causative factors of the GRβ expression is of significance. It has been recognized that TSLP is an alarmin cytokine in the initiation of Th2 response and allergic disorders 20. TSLP is involved in the pathogenesis of severe asthma, one of the common conditions of CR, that can be attenuated by administration of anti-TSLP antibodies 21. Exposure to IL-2, IL-7 and TSLP can induce group 2 innate lymphoid cells and natural helper cells to express Bcl-xL and facilitate the CR development 12, 22. The present study expands the above findings by showing that exposure to TSLP can induce the GRβ expression in Eos and neutrophils in the airway tissues by both *in vivo* and *in vitro* experiments. Eos and neutrophils with high GRβ expression showed inadequate response to steroids as shown by the present data.

It is known that epithelial cells can produce TSLP. Thus, it is of interest to elucidate the regulation of TSLP production. Exposure to protease allergens can induce epithelial cells to release TSLP via activating PAR2 (protease-activated receptor-2) 23. Viral infection can induce epithelial cells to produce TSLP through activating the Toll-like receptor 3- interferon-related factor-3 pathway 24. One of the Th2 cytokine, IL-13, also induces TSLP production by epithelial cells 11. In line with these pioneer works, we induced TSLP production by airway epithelial cells. Importantly, we found that livin is required in the IL-13-induced TSLP production by epithelial cells as shown by depletion of livin abolished the TSLP production. Livin is one of the apoptosis inhibitors; it plays a role in blocking the caspase activities to interfere with the apoptosis machinery 25, and thus, promotes cancer cell growth. Livin can modulate dendritic cell properties to up regulate CD8^+^ cytotoxic T cells' anti-tumor capacity 8, indicating that livin has immune regulatory functions. The present study expands this knowledge slut by showing that livin is involved in TSLP production by airway epithelial cells.

To date, although it has been recognized that high TSLP expression is one of the important cytokines to initiate skewed Th2 responses and plays a critical role in the pathogenesis of allergic diseases 26, how the TSLP expression is sustained at high levels in epithelial cells remains elusive. The present data demonstrate that livin expression and Ras activation form a circuit in airway epithelial cells. Ras activation promotes the livin expression in epithelial cells; livin in turn binds to GAP1 to prevent Ras deactivation. Therefore, livin and Ras activation mutually potentiate each other to sustain the TSLP production in epithelial cells. Since TSLP plays an important role in CR, livin is required in the TSLP production, it is conceivable to block livin may attenuate or inhibit CR. Indeed, we observed that depletion of livin in epithelial cells blocked the GRβ induction in Eos and neutrophils of the airway tissues as well as inhibited experimental airway allergy and CR.

The limitation of this study is that the CR was only could be reproduced in an animal model. Prospect studies may focus on modulating the expression of livin in airway epithelial cells to suppress the CR status.

In summary, livin is associated with the CR development through promoting TSLP production by epithelial cells. In cooperating with Ras activation, livin is also involved in sustaining the high TSLP expression in epithelial cells that contributes to CR development and CR sustaining. Inhibition of livin attenuates experimental airway allergy and blocks the development of CR.

## Supplementary Material

Supplementary methods, figures and tables.

## Figures and Tables

**Table 1:** Demographic data of human subjects

	HC (*n* = 20)	CS (*n* = 20)	CR (*n* = 20)
Age, mean ± SD (year)	32.5 ± 5.5	35.5 ± 6.3	34.4 ± 7.8
Gender (male and female)	10 and 10	12 and 8	11 and 9
Asthma history	0	0	0
Aspirin intolerance	0	0	0
Nasal polyp in both sides	0	20	20
Nasal allergy	0	20	20
Chronic rhinosinusitis	0	20	20
Nasal surgery history	0	0	0
Blood Eos, mean ± SD, (10^9^/L)	0.12 ± 0.15	0.305 ± 0.11	0.486 ± 0.15*
Serum total IgE (kU/L)	25 (0-31)	122 (55-336)	143 (46-274)
Serum mite specific IgE (kU/L)	11 (0-18)	32.3 (11.5-54.4)	38.5 (15.6-62.5)
Mite antigen SPT positive	0	20	20

*p<0.05. Mite: Indicate both *D. farinae* and *D. pteronyssinus*. Both total IgE and specific IgE were determined by ImmunoCap. Eos: Eosinophils. HC: Healthy control. CS: Corticosteroid sensitiveness. CR: Corticosteroid resistance.
