# Supplementary material for: Livin in synergy with Ras induces and sustains corticosteroid resistance in the airway mucosa
Source: Int J Biol Sci. 2021 May 13;17(8):2089–98. doi: 10.7150/ijbs.58427 (PMC8193260; doi:10.7150/ijbs.58427)
Supplement: Supplementary file 1 — Supplementary methods, figures and tables. [file ijbsv17p2089s1.pdf]

Supplementary materials

Reagents

Farnesyl thiosalicylic acid (FTS), Abs of pan Ras (C-4), CD4 (MT310; Alexa Fluor® 488), EpCAM (EBA-1; Alexa Fluor® 594), CD11b (44; Alexa Fluor® 488), livin (E-3), HRP-His tag (H-3), pan-cytokeratin (80), Ly-6G Ab (RB6-8C5; Alexa Fluor® 594), IL-13 receptor α1 (D-2) and Rac GAP1 (A-6) were purchased from Santa Cruz Biotech (Santa Cruz, CA). Abs of siglec F-PE and CD66b-APC were purchased from BD Bioscience (Franklin Lakes, NJ). PE-Siglec 8 Ab was purchased from Biolegend (San Diego, CA). Anti-TSLP Ab (ab188766), ELISA Kits of Ras GTPase and Ras GDP were purchased from abcam (Cambridge, MA). Recombinant TSLP, livin, GAP and Ras were provided by Shanghai Sangon Biotech (Shanghai, China). GRβ (PA3-514), GRα (PA1-516) Abs, reagents and materials for RT-qPCR and Western blotting were purchased from Invitrogen (Carlsbad, CA). ELISA kit for determining IL-4, IL-5, IL-13, ECP, NE and TSLP was purchased from R&D Systems (Minneapolis, MN). ELISA kit of ovalbumin-specific IgE was purchased from Biocompare (South San Francisco, CA).

Isolation single cells from nasal tissues

Surgically removed nasal mucosal tissues were collected from operation facilities of our hospital. The tissues were cut into small pieces, incubated with a solution containing 1 mg/ml collagenase IV and 0.05% ethylenediaminetetraacetic acid (EDTA) for 30 min at 37 °C with constant mild agitation. Single cells were passed through a cell strainer (100 µm first, then 70 µm) and further separated into epithelial cells and mononuclear cells by the Percoll gradient density centrifugation. Cell viability was greater than 99% as assessed by the Trypan blue exclusion assay.

Live nasal epithelial sample collection

Before the corticosteroid test, nasal epithelial samples were collected from each human subject by scrapping the inferior nasal turbinate surface with a plastic curette. The samples were then processed immediately for RT-qPCR analysis.

Purification of NECs, eosinophils (Eos) and neutrophils by flow cytometry (FCS)

NECs, Eos and neutrophils were further purified from the single cells described above by FCS. Briefly, single cells were labeled with Alexa Fluor® 594-EpCAM Ab for NEC purification, Alexa Fluor® 488-CD11b Ab + PE-siglec F Ab (for mouse Eos), Fluor® 488-CD11b Ab + PE-siglec 8 Ab (for human Eos), Alexa Fluor® 488-CD11b Ab + Alexa Fluor® 594-Ly6G Ab (RB6-8C5) Ab (for mouse neutrophils), Fluor® 488-CD11b Ab + APC-CD66b Ab (for human neutrophils), and then, purified by FCS. Purity of isolated cells was checked with FCS. All isolated cell purity was greater than 95%.

Primary NEC culture

Following published procedures (Forrest IA, Murphy DM, Ward C, Jones D, Johnson GE, Archer L, Gould FK, Cawston TE, Lordan JL, Corris PA. Primary airway epithelial cell culture from lung

transplant recipients. Eur Respir J. 2005 Dec;26 (6): 1080-5) with minor modification, purified NECs were cultured in Corning® Transwell® polycarbonate membrane cell culture inserts (Milipore Sigma) containing basal epithelial growth medium (Cambrex, San Diego, CA, USA) supplemented with BEGM™ Single quots (Cambrex), penicillin 50 U/ml, streptomycin 50 µg/ml, gentamicin 50 µg/ml and amphotericin B 50 ng/ml. The plates were placed in a CO_2 incubator (37°C/5% CO_2).

Assessment of Ras activation in airway epithelial cells
NECs were lysed with a lysis buffer (1.5 mM $MgCl_2$; 0.5 mM DTT; 10 mM KCl; 1 mM EDTA; 10 mM HEPES; 0.05% NP40 and protease inhibitor cocktail). Preexisting immune complexes in the lysates were eliminated by incubating with protein G sepharose beads for 2 h at 4 °C. The beads were removed from the samples by centrifugation at 13,000 *g* for 5 min. Supernatant was collected and incubated with an anti-Ras Ab at a final concentration of 1 µg/ml for 2 h at 4 °C to form the Ras/anti-Ras Ab complexes. The complexes were precipitated by incubating with protein G sepharose beads for 2 h at 4 °C. The beads were harvested by centrifugation at 13,000 *g* for 5 min. Proteins on the beads were eluted with an eluting buffer (10 mM Tris-Cl, 1 mM EDTA, pH 8.0). The proteins were added to a $TrisPO_4$/EDTA/DTT solution and heated to 100°C for 3 min to separate GTP and GDP from the immunoprecipitated Ras protein. The GTP and GDP levels in the samples were determined by the GTP ELISA and GDP ELISA, respectively, with commercial reagent kits following the manufacturer's instructions. The formula, Ras-bound GTP/(Ras-bound GTP + Ras-bound GDP) × 100 and was measured as described previously {Scheele, 1995 #38}, was used to calculate the Ras activation status.

Assessment of livin binding GAP1 with competitive ELISA
Recombinant (r) Ras, rGAP1 and rlivin proteins were provided by the Sangon Biotech Inc. (Shanghai, China). Microplates were coated with rRas in a coating buffer (anhydrous $NaHCO_3$, 2.93 g; anhydrous Na_2CO_3, 1.5 g; distilled water, 1 liter; pH to 9.6) (100 µl/well; 10 µg/ml). The plate was incubated at 4 °C overnight, washed with wash buffer (PBS containing 0.05% Tween-20) 3 times, blocked by adding blocking solution (PBS containing 1% w/v BSA) 150 µl and incubated at 37 °C for 1 h. A mixture of rGAP1-His and rlivin (diluted to 1:50, 1:100, 1:200, 1:400, 1:800, 1:1600 and 1:3200, respectively) was added to plate at 100 µl/well, incubated at 37°C for 1 h and washed with wash buffer 3 times. An anti-His Ab (Peroxidase-conjugated; diluted in 1:5000) was added to each well at 100 µl/well, and incubated at 37°C for 1 h, washed with wash buffer 3 times. Tetramethylbenzidine (TMB) was added to the plate at 100 µl/well, incubated for 30 min at room temperature. The reaction was terminated by adding 50 µl of H_2SO_4 (0.2 M). The absorbance was recorded with a microplate reader (BioTek™ Synergy™ HTX Multi-Mode Microplate Reader; Thermo Fisher Scientific) at 450 nm.

Mass spectrometry (MS)
NEC protein extracts were precipitated by anti-livin antibody. The precipitated proteins were sent to MS facility to be analyzed by MS. MS procedures were carried out by professional MS staff. MS data were processed by MS staff.

Treating airway allergy mice with dexamethasone (Dex)

A group of TR mice and a group of CR mice were treated with Dex at each time of OVA exposure by mixing Dex in OVA solution. The final Dex concentration was (5 mg/ml).

Mice

BALB/c mice (6-8-week-old) were purchased from the Beijing Experimental Animal Center (Beijing, China). Mice with livin-deficient epithelial cells were developed by the Beijing Agricultural Animal Institute (Beijing, China. Also see below). CreER^{T2} mice were purchased from Taconic Biosciences (Rensselaer, NY). Mice were maintained in a specific pathogen-free animal facility at Shanxi Medical University and were allowed to access food and water freely. The animal experiments were approved by the Animal Ethical Committee at Shanxi Medical University (#SMUETHAM2018002).

Mouse BALF collection

Immediately after the sacrifice, a 22 G angiocatheter (attached with a 1-ml syringe) was inserted into the trachea; 1 ml of PBS was instilled into the lungs through the angiocatheter. The BALF was recovered by slowly withdrawing the syringe plunger. The collection was repeated 2 more times for each mouse. BALF from 3 collections was pooled.

Protein extraction from cells

Cells were lysed with a lysis buffer (1.5 mM $MgCl_2$; 0.5 mM DTT; 10 mM KCl; 10 mM HEPES; 0.05% NP40; 1 mM EDTA and protease inhibitor cocktail). Lysates were centrifuged at 13,000 *g* for 10 min. Supernatant was collected and saved as the cytosolic proteins. The pellets were resuspended in a nuclear lysis buffer (0.2 mM EDTA; 1.5 mM $MgCl_2SO_4$; 4.6 M NaCl; 0.5 mM DTT; 5 mM HEPES; 26% glycerol) and incubated for 30 min. Lysates were centrifuged at 13,000 *g* for 10 min. Supernatant was collected and saved as the nuclear proteins. All the procedures were performed at 4 °C.

Flow cytometry (FCS)

In the surface staining, cells (10^6 cells/sample) were incubated with fluorochrome-labeled Abs of interest (1:100 dilution) or isotype IgG on ice for 30 min. After washing with phosphate-buffered saline (PBS), cells were analyzed with a flow cytometer (FCSCanto II, BD Bioscience). In the intracellular staining, cells were firstly fixed with 1% paraformaldehyde for 1 h, washed with PBS 3 times, and then processed with the same procedures of the surface staining. The data were analyzed with Flowjo (Tree Star Inc., Ashland, OR). The data of isotype IgG staining were used as a gating reference.

Real-time quantitative RT-PCR (RT-qPCR)

RNA was extracted from cells (NECs, Eos, neutrophils) with TRIzol reagents and converted to cDNA with a reverse transcription kit according to the manufacturer's instruction. The cDNA was amplified in a qPCR device (CFX96 Touch qPCR Detection System) with SYBR Green Master Mix and in the presence of livin primers (homo: agaggaggaagaggaggagg and

acctcaccttgtcctgatgg) or TSLP (Homo: acaacttgtagggctggtgt and aacatttctttggcgagcga; Mus: agcctcttcatcctgcaagt and cttgctctcacagtcctcga), GRα (Homo: acgaactagctgcccatctt and accagaaaagggaagttgtgc; Mus: tagggagagggagctggg and catggtcggatgaggatggt), GRβ (Homo: acctggggtcactaatcctg and ttttgtgtgtgtgtctggtttt; Mus: cgggttctcactggtacaca and tctcaaaggatggtgggact) and IL-13 receptor α (gccatcgagaagacccagag and tgaactgtccctcgcgaaaa). The results were calculated with the $2^{-\Delta\Delta Ct}$ method and presented as relative quantification (RQ).

RNA sequencing
Nasal epithelial cells (NEC) were collected from surgically removed nasal mucosa and purified by FCS with EpCAM as the NEC marker. Total RNA was extracted from NECs with TRIzol reagents and sent to the EGene Biotech company to be analyzed by RNA sequencing. The data were processed by bioinformatic staff of the company. Briefly, the RNA sequencing libraries were prepared by using an RNA library kit (TruSeq RNA library prep kit v2; Illumina). Libraries were sequenced using an Illumina HiSeq 2500 sequencer. RNA-seq read quality was assessed using FastQC. The activities of differentiated expressed gene (DEG) were reported as a heatmap.

Enzyme-linked immunosorbent assay (ELISA)
Levels of serum OVA-specific IgE, cytokines, mouse eosinophil cationic protein (ECP) and mouse neutrophil elastase (NE) in BALF or serum were determined by ELISA with commercial reagent kits following the manufacturer's instruction.

Immune cell culture
Immune cells were cultured with RPMI1640 medium that was supplemented with 2 mM glutamine, 10% fetal bovine serum, 100 U/ml penicillin and 0.1 mg/ml streptomycin. For Eo culture, GM-CSF at 1 ng/ml was added to the culture. NECs [(a human nasal epithelial cell line) were purchased from PromoCell (Heidelberg, Germany)] were cultured in DMEM (Dulbecco's Modified Eagle Medium) supplemented with 2 mM glutamine, 10% fetal bovine serum, 100 U/ml penicillin and 0.1 mg/ml streptomycin. Cell viability was greater than 99% as assessed by the Trypan blue exclusion assay.

Western blotting
Proteins were extracted from experimental cells, fractioned by sodium dodecyl sulphate-polyacrylamide gel electrophoresis (SDS-PAGE) and transferred onto a PVDF (polyvinylidene fluoride) membrane. After blocking with 5% skim milk for 30 min at room temperature, the membrane was incubated with primary antibodies (diluted to 1:500) overnight at 4 °C, washed with Tris-buffered saline containing 0.05% Tween 20 (TBST) 3 times, incubated with secondary antibodies (labeled with HRP; diluted to 1:5000) for 2 h at room temperature. Immunocomplexes on the membrane were developed by the enhanced chemiluminescence and photographed in the GelDoc Go image station (Bio-Rad).

Co-immunoprecipitation (co-IP)
Proteins were extracted from cells as described above and incubated with protein G agarose beads for 2 h to eliminate pre-existing immune complexes from the samples, followed by centrifugation at 5,000 *g* for 5 min to remove the beads. Supernatant was collected and

incubated with Abs of interest at 1 μg/ml or isotype IgG overnight; the samples were incubated with protein G agarose beads for 2 h to precipitate the immune complexes in the samples; the beads were collected by centrifugation at 5,000 *g* for 5 min. Proteins on beads were eluted with an eluting buffer (10 mM Tris-Cl, pH 8.5) and analyzed by Western blotting.

Assessment of airway resistance

Airway resistance was tested in each mouse one day after the last antigen challenge. Mice were peritoneally injected with 5% chloral hydrate at 400 mg/kg to be anesthetized. Tracheal intubation was performed on each mouse, mice were placed in a closed body drawing box and ventilated mechanically. Mice were nebulized with methacholine at 0.1 ml containing gradient concentrations of methacholine (0, 6.25, 12.5, 25, and 50 μg/ml). After inhalation, data from 5 seconds to 1 minute were acquired, from which the lung resistance was calculated; the results were presented as fold change.

Generation of mice carrying livin-deficient epithelial cells

The livin gene in epithelial cells were depleted in BALB/c mice by genetic engineering approaches. To avoid influencing the role of livin in other cells, the livin gene was only deleted from epithelial cells with EpCAM as the specific marker. Following reported procedures (J Clin Invest 1996; 98: 600-3; Elife 2014; 3: e01949), mice with loxP-flanked livin gene (flox) were developed. A gene targeting vector containing three loxP sites was constructed, in which the neomycin resistance gene was flanked in two of them. By homologous recombination, the genomic locus was modified between vector and the livin gene in embryo stem (ES) cells. The loxP-flanked livin gene was targeted by EpCAM-Cre expression in ES cells. Using the modified ES cells, a loxP-livin containing mouse line was generated. Then, the mouse strain carrying two loxP sites in the livin gene was crossed with another strain expressing EpCAM-Cre recombinase. Thus, only in cells expressing EpCAM-Cre the livin gene becomes inactivated but remained active in other cells of the body. To keep $EpCAM^+$ cell active until the experimental period, the Cre gene was modified to be functional only in the ribosomes, but could not penetrate the nuclear membrane. Therefore, the loxp-livin gene was remained intact in the mice. Five days before the experiments, the mice were treated with tamoxifen citrate salt (Sigma Aldrich, St. Louis, MO; 0.3 g/kg in 0.2 ml 10% Etoh in corn oil) via gavage-feeding daily for five consecutive days. Control mice were received oral gavage of 0.2 ml 10% EtoH in oil. Tamoxifen iCSeases the permeability of the nuclear membrane to allow the EpCAM-Cre penetrate into the nuclei to cleave the flox-livin gene. As assessed by RT-qPCR and Western blotting, all $EpCAM^+$ epithelial cells in the airway tissues did not express livin. The livin gene knockout effects are presented in Fig. S1B-E (see below).

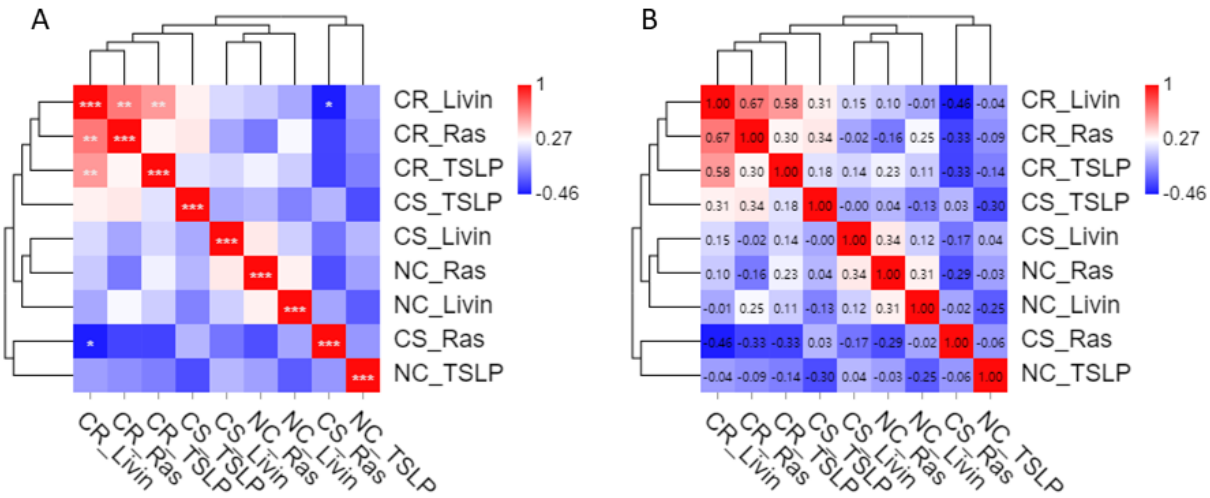

Figure S1. Correlationship between livin, Ras and TSLP in nasal epithelial cells. Spearman correlation test was performed with the data of livin, Ras and TSLP in nasal epithelial cells within groups of the NC, or AR_CS, or AR_CR as shown in Fig. 1. The heatmaps show the significant levels of coefficient (A) and the individual coefficient (B). *, p<0.05; **, p<0.01; ***, p<0.001.

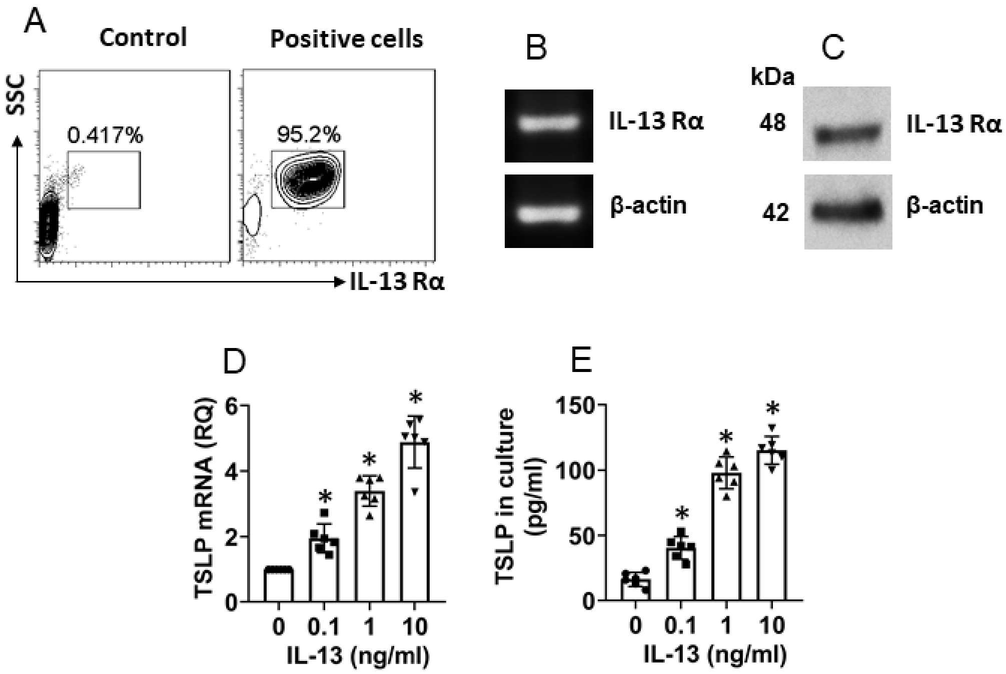

Figure S2. IL-13 induces nasal epithelial cells to produce TSLP. Normal NECs were isolated from surgically removed nasal tissues of patients with nasal cancer (n=6). The adjacent normal nasal tissues were isolated (proved by a pathologist) to be used in the experiments. The NECs were analyzed by FCS, RT-PCR and Western blotting. A, gated FCS plots show IL-13 receptor α (IL-13Rα)$^{+}$ cell counts. B, IL-13Rα mRNA was detected in NECs. C, IL-13Rα protein was detected in

NECs. D-E, NECs were cultured at 10^6 cells/ml in the presence of TSLP at indicated concentrations for 48 h. Cells were analyzed by RT-qPCR. Supernatant was analyzed by ELISA. D, bars indicate the TSLP mRNA levels in NECs. E, bars indicate the TSLP levels in culture supernatant. Statistics: ANOVA followed by the Dunnett's test.

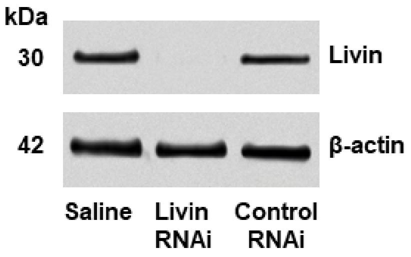

Figure S3. Depletion of livin expression in NECs. NECs were treated with RNAi reagents of livin or control RNAi reagents with commercial reagent kits following the manufacturer's instruction. The cells were analyzed 48 h later. Immunoblots show livin protein in NECs. The data represent 3 independent experiments.

Livin protein amino acid sequence

MGPKDSAKCLHRGPQPSHWAAGDGPTQERCGPRSLGSPVLGLDT
CRAWDHVDGQILGQLRPLTEEEEEEGAGATLSRG**PAFP**GMGSEELRLASFYDWPLTAE
VPPELLAAAGFFHTGHQDKVRCFFCYGGLQSWKRGDDPWTEHAKWFPSCQFLLRSKGR
DFVHSVQETHSQLLGSWDPWEEPEDAA**PVAPSVP**ASGY**PELP**TPRREVQSESAQEPGA
RDVEAQLRRLQEERTCKVCLDRAVSIVFVPCGHLVCAECAPGLQLCPICRAPVRSRVRTFLS

Figure S4. Prolines in livin protein amino acid sequence. The amino sequence of proline shows the "PxxP" structures (highlighted in red).

Peptide 1

Amino acid sequence:
(414) VPLLSKVDDIHAICS (428)

Gene sequence:
(1216) Gtacccctcctcagcaaagtggatgatatccatgctatctgtagc (1260)

Peptide 2

Amino acid sequence:
(585) LLGPVTTPEHQLLKT (600)

Gene sequence:
(1720) Ttactgggacctgtgaccactcctgaacatcagcttctcaagact (1764)

Figure S5. Amino acid sequence and gene sequence of Rac GAP1 in livin Ab-precipitated complexes. Protein extracts were prepared with CR nasal tissue samples (n=3). The proteins were precipitated with anti-livin Ab as a bait. The precipitated complexes were analyzed by mass spectrometry. The presented two representative peptides indicate the precipitated protein binding livin is Rac GAP1 (GenBank: LS482413.1). The numbers in () are start and end sites in the sequence. The data represent 3 independent experiments.

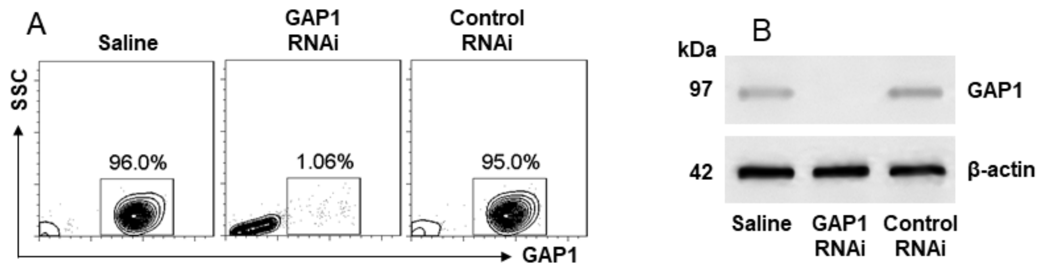

Figure S6. Depletion of GAP1 expression in NECs. NECs were treated with GAP1 RNAi reagents or control reagents following the manufacturer's instructions. The cells were harvested 48 h after the transfection. GAP1 expression in the cells were assessed by FCS and Western blotting. A, gated FCS plots show $GAP1^+$ HNEpC counts. B, immunoblots show GAP1 protein in NECs. The data represent 3 independent experiments.

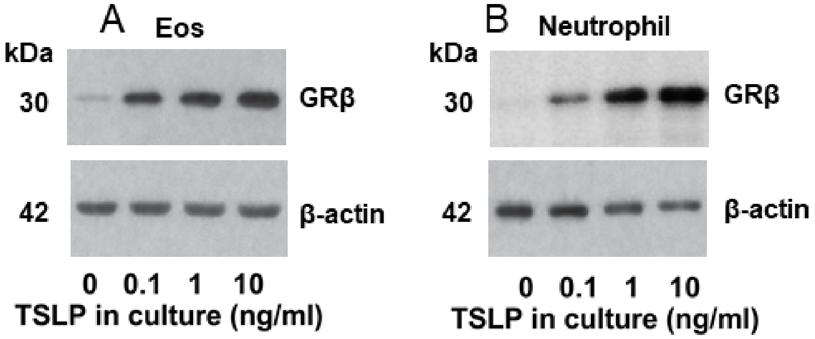

Figure S7. TSLP induces naive Eos and neutrophils to express GRβ. Eos and neutrophils were isolated from peripheral blood of healthy subjects; cells were cultured (10^6 cells/ml) in the presence of TSLP at indicated concentrations for 48 h. Cells were harvested at the end of culture and analyzed by Western blotting. The immunoblots show GRβ protein levels in protein extracts of Eos and neutrophils. The data represent 3 independent experiments.

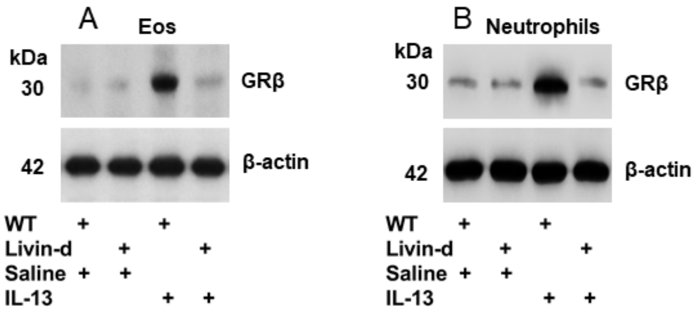

Figure S8. Inhibition of livin blocks the GRβ expression in Eos and neutrophils in the airway tissues. Naive mice were treated with IL-13 solution (50 µg/ml) via nasal instillation (50 µl/nostril) daily for 7 consecutive days. Mice were sacrificed on day 8. Airway tissues (including the nasal mucosa and the lungs) were excised. Eos and neutrophils were isolated from the tissues. Protein extracts were prepared from Eos and neutrophils and analyzed by Western blotting. The immunoblots show GRβ protein levels in protein extracts of Eos and neutrophils. The data represent 3 independent experiments.

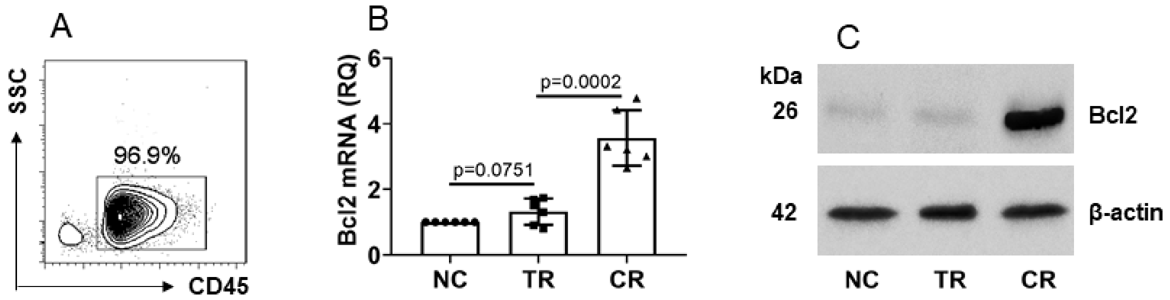

Figure S9. Assessment of Bcl2 expression in immune cells isolated from the mouse airway tissues. Single cells were isolated from the mouse airway tissues. $CD45^+$ cells were further isolated from the single cells by FCS. A, gated FCS plots show cell purity of isolated $CD45^+$ cells. B, bars show Bcl2 mRNA levels in isolated $CD45^+$ cells. C, immunoblots show Bcl2 protein levels in isolated $CD45^+$ cells.

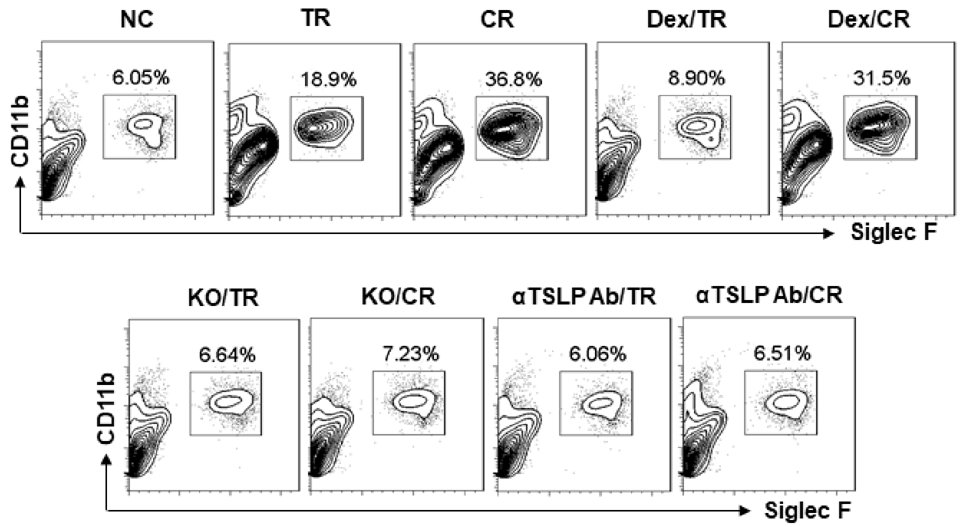

Figure S10. Eo counts in BALF. Supplementary data for Fig. 6I. Gated FCS plots show representative Eo counts in BALF.

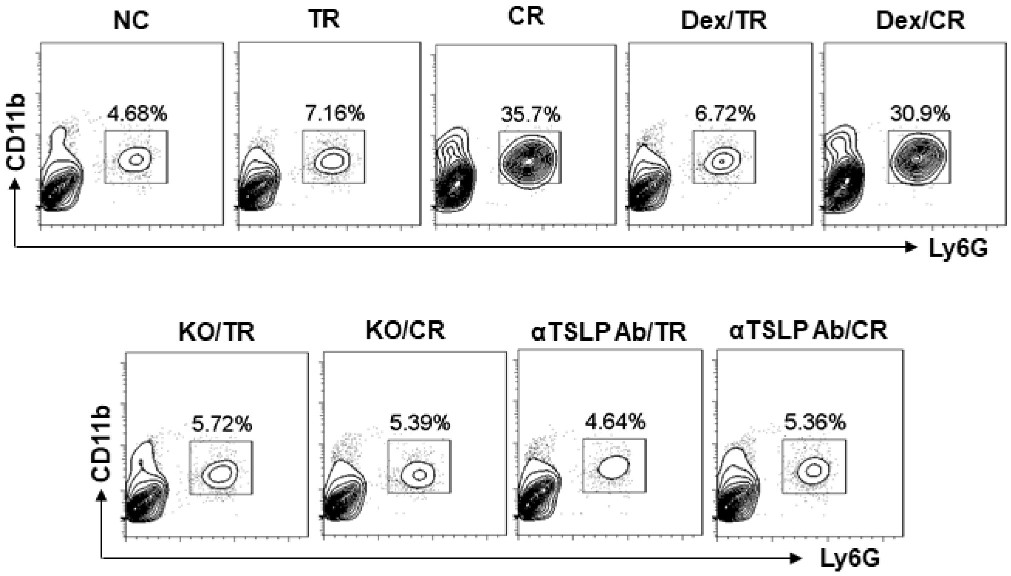

Figure S11. Neutrophil counts in BALF. Supplementary data for Fig. 6J. Gated FCS plots show representative neutrophil counts in BALF.

Table S1. Positive allergens

Allergens	mites	molds	cockroach	dog	cat	timothy	oak	maple	ragweed
CRSwNP	20	5	3	4	3	3	6	6	5
%	100	25	15	20	15	15	30	30	25
CRSsNP	20	6	4	4	4	3	4	3	3
%	100	30	20	20	20	15	20	15	15

CRSwNP: Chronic rhinosinusitis with nasal polyp. CRSsNP: Chronic rhinosinusitis without nasal polyp.

Table S2. Polyp scores of each subject

Groups	prednisone use	Scores																			
CS	Prior	2	4	4	3	3	3	4	4	2	4	3	4	2	2	2	4	4	3	3	2
	Post	1	2	2	2	2	2	2	2	1	3	2	2	2	3	1	2	2	2	2	1
CR	Prior	2	3	4	4	2	3	3	2	4	3	4	3	3	3	4	3	2	2	3	3
	Post	3	3	4	4	4	3	3	3	4	3	4	3	3	3	4	3	3	2	3	3

CR: Patients with corticosteroid resistance; CS: Non-CR patients;
